# Mouth Breathing

**Published:** 1899-07

**Authors:** James M. Crawford

**Affiliations:** Atlanta, Ga.


### SELECTIONS.

### Mouth Breathing.

BY JAMES M. CRAWFORD, M.D., ATLANTA, GA.

Read before the Medical Association of Georgia, Macon, April 19-21, 1899.

My paper has reference to those cases where mouth-breathing is the rule and not the exception. If one will take the pains to minutely examine the interior of the nose and its function, he will not be surprised to learn that there is a penalty for those who persistently use the mouth as a breathing apparatus. The object of nasal breathing is twofold—to moisten and to warm the air before its reception into the bronchial tubes. We need not be surprised, then, if many cases of bronchitis are caused by mouth-breathing, the cold and dry air coming in contact with the bronchial tubes and air-cells, causing congestion, and later inflammation. The same condition often causes asthma. We no longer attribute mouth-breathing to habit, and scold the patient for not breathing through the nose, but on the contrary, we know that in these cases there is an obstruction in the nares, postnasal or pharyngeal spaces that forces one to breathe through the mouth instead of through the proper channel—the nose.

The most usual causes of nasal obstruction are the various forms of spurs and deflections of the septa, and now and then cases of polypi, and hypertrophies of the turbinates. Usually, accompanying these conditions, we have adenoids in the post-nasal space and frequently hypertrophied tonsils; indeed, so universally is this the case that I consider the examination incomplete where one does not examine very closely for the other conditions just mentioned, having already found that one exists. These various obstructions are generally met with in childhood or young manhood or womanhood, from the fact that the nasal, as well as the pharyngeal spaces, are much smaller at this age, and as they grow older these spaces are made larger, tending thereby toward relieving the patients, at any rate making them more comfortable. The frequency of these cases, especially of adenoids, met with here in the South shows that they are as numerous as in the North or West, and that a cold climate is not needful for their development. I am satisfied that adenoids are often hereditary, and am therefore very careful with my examinations of a family when I have found one of them suffering from this trouble. We often see children of an entire household similarly affected. No race seems to be exempt. I have seen and operated upon both white and colored; indeed, some of the worst cases of adenoids I have ever met with were in the colored race. The reason we see so few in the colored race is because of their financial inability to have such work done, and therefore they do not come to us.

The diagnosis in these cases is very easily made, as the pharyngeal tonsil can readily be examined. By placing a little mop of cotton dipped in a 6 percent solution of cocain muriate in the nasal spaces, we are enabled to discover the hypertrophied turbinates, as this application has the happy power of relieving engorged tissue, thereby making the diagnosis sure. I find cocain to be of great aid in the diagnosis of such cases, and I never fail to use it. The use of cocain, however, should be limited to making such examinations, and in cases where some form of operation is to be made. It should not be used habitually in the treatment of catarrhal conditions. The rhinoscope is of comparatively no use in diagnosing a case of adenoids, since but few patients can be trained to allow a mirror to be used in the mouth so as to make it possible to see anything in the postnasal spaces. Educate the sense of touch so as to be able to detect a case of adenoids by the use of the finger, which can readily be done. Not only can the adenoids be detected, but by this means you are enabled to say to what mode of operation you will resort. If soft and easily broken down, I prefer the use of my finger-nail; if more fibrous, I prefer the Gleitsmann forceps. The causes of these various obstructions are but little known. To say they are hereditary traits is not making it plainer to the inquiring mind. There is no class of patients for whom the doctor can do more than for these.

To relieve one of these obstructions is to place new life in the patient, to make him happier, to make him less liable to the various forms of ear trouble that sooner or later most usually develop, and to make less liable the bronchial affections, asthma, etc. The successful restoration of nasal breathing, then, depends upon the removal of these obstructions. Spurs and deflections of the septum can be removed best by the saw. I prefer a thin-bladed and therefore a fine-toothed saw to cut when pushing from, and the other when pulling toward the operator—the former to be used when the obstruction is situated on the middle or near the front portion of the septum, the latter when the obstruction is at the rear end of the septum. No anesthetic is needful other than the use locally of a 10 percent solution of cocain muriate. The same method of local anesthesia is sufficient in reducing hypertrophies of the turbinates, with either the cautery or chromic acid. The idea should be not to burn the whole surface of the turbinate, but simply to burn the most projecting portion quite deeply, and in this way to cause a contraction of the tissue—buttoning the turbinate down as it were.

I have tried removing the adenoids in many ways, but have found none to equal that with the Gleitsmann forceps when the adenoids are fibrous, and with the finger when of the soft variety. I prefer to do this without an anesthetic, since statistics show the death of so many when made under the influence of chloroform or ether. The operation is not a painful one and is quickly made. It is not needful to put the patient to bed either before the operation or after, as very little reaction follows. I have never seen much hemorrhage follow the operation. I have made many operations on children as young as 4 years without any resistance whatever and without even the patient crying. I admit, however, that the temperament of the patient has much to do with it, some bearing pain much better than others. The operation sometimes has to be repeated.—*Jour. Am. Med. Asso.*

